# Simple imputation method for meta-analysis of survival rates when precision information is missing

**DOI:** 10.1017/rsm.2025.10024

**Published:** 2025-09-11

**Authors:** Kazushi Maruo, Yusuke Yamaguchi, Ryota Ishii, Hisashi Noma, Masahiko Gosho

**Affiliations:** 1 Department of Biostatistics, Institute of Medicine, University of Tsukuba, Ibaraki, Japan; 2 Biostatistics, Data Science, Astellas Pharma Global Development, Inc., Northbrook, IL, USA; 3 Department of Data Science, The Institute of Statistical Mathematics, Tokyo, Japan

**Keywords:** confidence interval, imputation, Kaplan–Meier method, R package

## Abstract

In meta-analyses of survival rates, precision information (i.e., standard errors (SEs) or confidence intervals) are often missing in clinical studies. In current practice, such studies are often excluded from the synthesis analyses. However, the naïve deletion of these incomplete data can produce serious biases and loss of precision in pooled estimators. To address these issues, we developed a simple but effective method to impute precision information using commonly available statistics from individual studies, such as sample size, number of events, and risk set size at a time point of interest. By applying this new method, we can effectively circumvent the deletion of incomplete data, resultant biases, and losses of precision. Based on extensive simulation studies, the developed method markedly improves the accuracy and precision of the pooled estimators compared to those of naïve analyses that delete studies with missing precision. Furthermore, the performance of the proposed method was not significantly inferior to the ideal case, where there was no missing precision information. However, for studies for which the risk set size at the time of interest was not available, the proposed method runs the risk of overestimating the SE. Although the proposed method is a single-imputation method, the simulations show that there is no underestimation bias of the SE, even though the proposed method does not consider the uncertainty of missing values. To demonstrate the robustness of our proposed methods, they were applied in a systematic review of radiotherapy data. An R package was developed to implement the proposed procedure.

## Highlights

### What is already known?

- It is difficult to handle studies with missing precision information when conducting a meta-analysis of survival rates.
- However, excluding such studies from a meta-analysis may significantly reduce precision and accuracy.

### What is new?

- We developed a method to impute precision information using information commonly available from the study literature, such as sample size, number of events, and risk set size at a time point of interest.
- An R package is provided to implement the proposed imputation method (Imp method).

### Potential impact for RSM readers

- When precision information is missing, the proposed method improves the precision and accuracy of meta-analyses of survival rates and leads to better medical conclusions.
- The given package is easily installable and facilitates implementation of the proposed method.

## 1 Introduction

In meta-analyses of clinical studies on survival outcomes, hazard ratios are often the target of estimation. A hazard ratio is generally estimated based on a Cox proportional hazards model^1^ and describes how many times more (or less) likely a participant is to suffer the event at a particular point in time if they receive the experimental rather than the comparator intervention.^2^ Hazard ratios can be integrated by calculating the log hazard ratios and their standard errors (SEs) from the reported hazard ratios and their confidence intervals (CIs)^3^ Hazard ratios are also sometimes estimated by reconstructing pseudo individual patient data (IPD) from published Kaplan–Meier curves.^4^^,^ ^5^ The Cochrane handbook states that the most appropriate way of summarizing time-to-event data is to express the intervention effect as a hazard ratio.^2^

In contrast, epidemiological studies often aim to summarize survival outcomes in a single population. Furthermore, in the field of rare diseases, conducting randomized controlled trials is often impractical, even for interventional studies with survival outcomes. In such cases, scientific decisions must rely on single-arm studies. Indices used in the meta-analysis of single-arm survival outcomes include survival rate, median survival time,^6^ and restricted mean survival time.^7^ In this study, we consider a situation in which the primary interest is a meta-analysis of survival rates at a specified time estimated using the Kaplan–Meier method in a single population.

Several methods have been developed for the meta-analysis of survival functions.^8^^,^ ^9^ Combescure et al.^9^ proposed a method to perform a meta-analysis of the survival function from the increment of the survival function at each event time point and its variance estimator by scanning the images of Kaplan–Meier plots of all studies and the risk set size at each time point. Computing the increments of the survival function at all event time points can be viewed as recovering pseudo-IPD data. In this study, we considered a scenario in which a single time point was the primary focus. Among the included studies, some reported the survival function along with its associated precision information (e.g., CIs), while others did not provide precision information, resulting in a mix of studies. It is desirable to make effective use of the available precision information from studies that report it, without employing the pseudo-IPD restoration method. In contrast, excluding studies that lack precision information may reduce overall precision and introduce bias. Therefore, to mitigate these issues, missing values should be imputed for studies without reported precision information.

In this study, we propose a simple method for imputing missing precision that requires not the entire Kaplan–Meier plot but only the total sample size, along with the risk set size and survival rate at the time of interest. Additionally, we developed a procedure for converting the results of studies that report precision information onto a unified scale, where meta-analysis is performed. Furthermore, the R package metaSurvMissCI was developed to implement the Imp method. Section 2 introduces several methods for estimating CIs for survival rates and provides a procedure for transforming reported precision information onto a unified scale. This section also describes a meta-analysis model of the survival rates. Section 3 describes the details of the proposed method in the case of missing precision information. Section 4 presents simulations to evaluate the performance of the proposed method, and Section 5 provides an example of real data, followed by discussion and summary in Section 6. A brief description of the developed R package is provided in the Appendix.

## 2 Meta-analysis for survival rate

### 2.1 Confidence interval for survival rate

Let 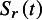 be the survival rate at time *t* for the *r*th study 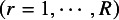 in the meta-analysis. The Kaplan–Meier estimator for 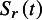 is denoted by 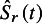 . There are three well-known methods for estimating CIs for survival rate: 1. delta method (Greenwood formula^10^), 2. log transformation method, and 3. log-log transformation method.^11^ These methods are based on asymptotic normality on a scale after transforming the survival rate by some function (including the identity transformation for the delta method). The variance estimators of the survival rates on the transformed scale for each method are: 

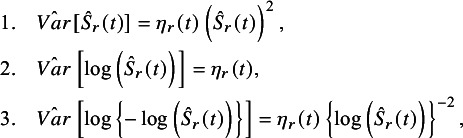

where (1)

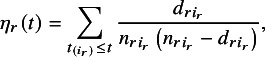




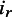
 is a subject index for the *r*th study 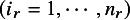 , 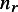 is the sample size for the *r*th study, 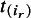 is a rank-order survival time, 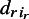 is the number of events that occurred at 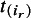 , and 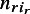 is the number at risk of event at 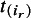 (see, e.g., Hosmer et al.^12^). CIs are constructed based on the asymptotic normality of the estimators using the variance estimators above. For example, the confidence interval for the log transformation method is 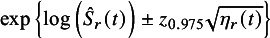 , where 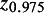 is the 97.5 percentile of the standard normal distribution. This study deals with 95% confidence intervals (CIs), which are commonly reported; however, other confidence levels are also available.

The default method for estimating CIs differs among statistical software packages. For example, the default method in the LIFETEST procedure of the SAS software (SAS Institute Inc., Cary, NC) is method 3, whereas the default method in the survival package^13^^,^ ^14^ of the R software (R Core Team, Vienna) is method 2. The syntax in the Web Help in SPSS software (IBM Corp., Armonk, NY) indicates method 1. (https://www.ibm.com/support/pages/kaplan-meier-survival-curve-confidence-limits).

Here, we consider which CI estimation methods should be applied to the meta-analysis, even though they are used differently among the studies. First, the delta method could easily lead to CI endpoints less than zero or greater than one. In addition, the assumption of normality implicit in the use of the procedure may not hold for small-to-moderate sample sizes, which is often seen in typical problems.^12^ The possible ranges of CIs for the log and log-log transformation methods on the transformed scale are 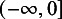 and 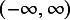 , respectively. Kalbfleisch and Prentice^11^ recommended the log-log transformation method because of these properties. Therefore, this study focused on the log-log transformation method as a unified scale for meta-analysis.

### 2.2 Procedure for transforming reported precision information into a unified scale

As the CI estimation method is expected to vary between studies, it is necessary to transform the point and variance estimates of all studies into a unified scale using the above formulas. In this section, we detail the a procedure for transforming reported precision information into a unified scale.

Consider the case in which a CI based on the log transformation method is transformed into an SE on a log-log transformation scale. First, the SE is calculated on the log-transformed scale as 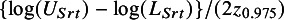 , where 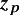 is the 100*p* percentile of standard normal distribution, and 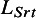 and 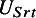 are the lower and upper confidence limits for 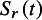 , respectively. As this value is equal to 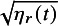 , the SE of the log-log transformation scale is then obtained as 
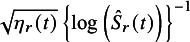
 .

For studies that report CIs, if the estimation method is specified in the article, the CIscan be transformed into SEs on a unified scale according to the method used; otherwise, estimation methods need to be estimated. The estimation method can be determined from the symmetry around the point estimate for the CI on the transformed scale. Specifically, we calculate 

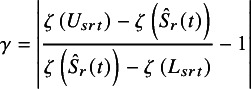

on the original scale, the log-transformed scale, and the log-log transformed scale, respectively, where 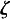 is a transformation function. For example, 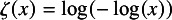 for the log-log transformation. Note that 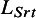 and 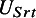 are reversed in the log-log method. Then, the scale that minimizes 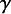 is determined as the estimation method. However, if the minimum value of the 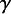 is far from 0 (e.g., exceeding 0.1), this may indicate an issue with the transcription from the article, an error in the reported value itself, rounding error caused by insufficient significant digits, or an unexpected method used for estimating the CI. In such cases, after verifying that the transcription from the paper is accurate, the precision information for the study should be treated as missing.

### 2.3 Meta analysis model

Let 
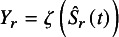
 be the survival function on an arbitrary transformed scale with the transformation function 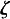 . We consider the following random-effects meta-analysis model: (2)

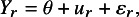

where 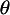 is the grand mean on the transformed scale, 
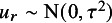
 is the random effect, and 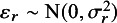 is the error term. 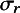 is the SE on the transformed scale for each study *r* as described in section 2.1. The unknown parameters are 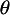 and 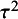 , which are usually estimated based on the Dersimonian–Laird method^15^ or restricted maximum likelihood (REML) method. Based on the either method, the point estimate and CI for 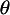 are obtained as 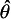 and 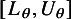 , respectively, where 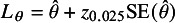 , 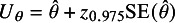 , and 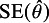 is the SE of 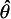 . The inverse transformation of 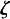 is applied to these results to obtain the point estimate and CI for the survival rate as 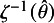 and 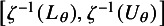 , respectively. Note that the CI is obtained as 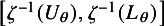 for the log-log transformation method because the log-log transformation is not order-preserving.

Under the assumption that 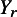 approximately follows a normal distribution, 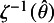 is regarded as the median estimator of 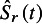 for any monotonic function 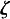 , owing to the symmetry of the normal distribution. If the distribution of 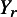 is not highly skewed and approximate symmetry is achieved, the median may be taken as the target estimand. Furthermore, if the true value of the survival function is not close to 1 or 0, the distribution of 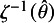 on the original scale is expected to be less skewed. In this case, the expected value and median of the survival function estimator would be close to each other, suggesting that the back-transformation of the meta-analysis results can be reasonably interpreted as the expected value.

It is difficult to define the true value of the estimator of a meta-analysis with a finite sample. In our simulations, we used the estimates from the meta-analysis when the number of studies is infinite (actually very large) and when there are no missing SEs as the true values.

## 3 Imp Method

Next, we discuss the imputation of missing CIs. Variance estimators can be calculated using either method if 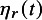 is available, even when precision information is missing. However, if IPD are unavailable, 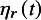 cannot be calculated directly. Therefore, we propose the following approximate formula when precision information is missing: (3)

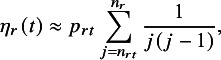

where *j* is a summation index, 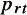 is a parameter related to the proportion of events in the number of individuals excluded from the risk set by time *t*, and 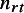 is the risk set size at time point *t*.

The natural estimator of 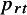 is 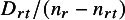 , where 
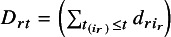
 ; that is, the cumulative number of events at time *t*. However, this information is usually not available from study articles. Information on 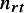 is sometimes obtained from Kaplan–Meier plots, where the risk set size at each time point is often shown below the survival curve. However, there may be studies in which 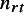 is not available.

Consider the following simultaneous equations to obtain the missing values of 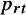 and 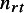 : (4)

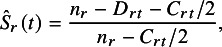


(5)

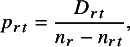


(6)

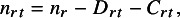

where 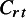 denotes the cumulative number of censored subjects at time *t*. Equation (4) approximates the Kaplan–Meier estimator using an estimator based on the life table method. Equation (6) is the definition. By eliminating 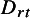 and 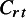 from these equations, we obtain: (7)

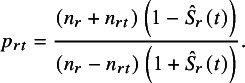



In studies where 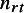 is available, 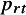 can be derived from the equation above. For studies, where 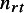 is not available, 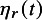 cannot be calculated. In such cases, 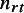 is first calculated based on the following equation, which is a transformation of Equation (7): (8)

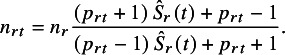

The right-hand side of the above equation contains 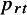 , which is typically unavailable. Therefore, 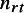 is calculated by substituting 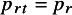 into Equation (8), where 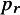 is the event occurrence proportion for the study *r*. As the number of events per study is reported in most cases, 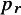 can be easily calculated. In studies in which 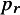 is not available, it may be necessary to substitute the mean value of 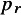 from other studies.

If 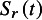 is estimated to be one, study *r* cannot be included in the meta-analysis because the SE becomes zero. When SE is 0, the weight of the study in question is infinite and the meta-analysis is not feasible. Moreover, for 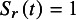 , the log-log transformation method yields a minus infinity, and the meta-analysis cannot be performed. Therefore, for convenience, we replace 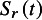 and its SE with 0.99 and NA (missing value), respectively. Moreover, if 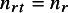 or 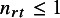 , SE also becomes 0 or 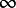 , respectively. Therefore, it is replaced by 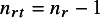 or 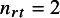 , respectively.

The proposed method relies on several assumptions:

1. There are no tie data; that is, all 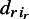 are 1 (Equation (3)).
2. The life table estimator approximates the Kaplan–Meier estimator (Equation (4)).
3. The effect of not adding a censored subjects in 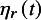 can be adjusted by multiplying the event proportion up to time *t* (Equation (5)).
4. Event occurrence rates before time *t* and during the entire period are the same (only when 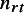 is not available).

Assumption 1 is not problematic because it can easily be shown that Equation (3) is equivalent to the original 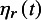 in Equation (1) when 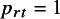 , that is, there is no dropout until time point *t*. In fact, it can be shown by the mathematical induction that 

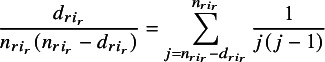

for any term on the right-hand side of Equation (1). Assumptions 2 and 3 require that the shape of the event and censoring hazard up to time *t* be similar. For example, if all the events occur after all the censoring has occurred, then Equation (4) becomes 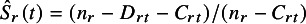 . As Equation (7) includes the Kaplan–Meier estimator, deviations from Assumption 2, that is, deviations of the life table estimator from the Kaplan–Meier estimator (referred to here as bias), lead to a bias of the SE. Specifically, over- and underestimation biases of the life table estimators lead to over- and underestimation of the SE, respectively. It is obvious that the deviation from condition 3, that is, the bias of 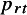 , leads directly to the bias of SE.

In addition, the proposed method is a single imputation method. In the context of missing measurements, the multiple imputation method is often used because the SE suffers from an underestimation bias if the uncertainty of completion is not considered based on the single imputation method. However, the situation in this study is different because the estimates themselves exist but only the SEs are missing.

The robustness of the proposed method with respect to these assumptions was evaluated through simulations.

## 4 Simulation study

### 4.1 Evaluation for meta analysis results

#### 4.1.1 Simulation design

In this section, we describe the simulations conducted to evaluate the performance of the Imp method for the meta analysis of survival rates. The distributions for event and censoring were set as 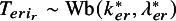 and 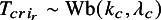 , respectively, where 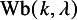 was a Weibull distribution with a shape parameter *k* and a scale parameter 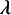 . We set the distribution of 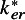 as follows: 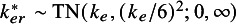 , where 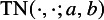 is a truncated normal distribution truncated at *a* and *b*, which means that approximately 99% of 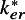 fell between 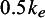 and 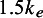 . This setting means that not all studies included in the meta-analysis had identical shape parameters (that is, heterogeneity). We used the truncated normal distribution because the shape parameter does not take negative values. However, the probability that 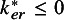 becomes almost zero, even if a normal distribution is used. We set 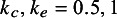 , or 2, which were intended to represent initial, accidental, and wear-out failure hazards. 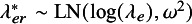 , where 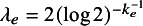 , which was intended such that the median survival time was 2 (years) when 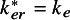 . The setting, whereby, 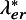 followed a lognormal distribution with variance parameter 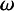 was intended for heterogeneity among the studies. More specifically, 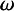 was set with the intention that the scale parameter for the Weibull distribution would vary among the studies. 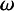 was numerically calculated so that the 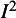 -statistic for the meta-analysis with the model (2) for 1,000 simulated studies ( 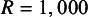 ) was 0%, 30%, or 60%. When 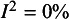 , we set 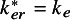 and 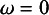 . When 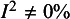 , the heterogeneity was induced by the distribution of 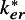 and 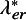 , but whether the value of 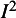 was 30% or 60% depended only on the value of 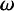 . It would be natural for the true values of the shape and scale parameters to vary among studies (i.e., to have heterogeneity). For example, it is possible that one study might have an initial failure hazard with a median survival time of 1.5 years, while another study might have an accidental failure hazard with a median survival time of 2 years. Although, we used 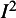 to set the value of heterogeneity parameter, 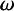 , 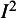 essentially measures the proportion of variability due to heterogeneity, not heterogeneity itself. 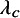 was numerically calculated such that the censoring proportion was 20% or 40%. We set the sample size for each study to 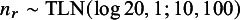 , which is a log-normal distribution truncated at 10 and 100. We set the number of studies to 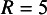 , 10, or 20. We also set situations relevant to the area of rare diseases, for which sample sizes are not very large.

For each study, the survival rate was estimated at 1, 2, and 3 years, and CIs were estimated using the log-log transformation method. For studies in which survival rate was not estimated at each time point (the maximum survival time was less than *t*), the estimates of the survival function were excluded from the meta-analysis, because the estimates of the survival function themselves would not be reported. The true value of the *t*-year survival rate for each condition cannot be explicitly determined because of the randomness of the Weibull distribution parameters. Furthermore, increasing 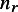 and *R* simultaneously alters the relationship between 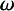 and 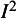 , and so we considered the case where *R* increased while keeping 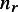 fixed. For this reason, estimates based on random numbers of 1,000 studies were used as true values. A meta-analysis incorporating data of a large number of studies, all of which reported SEs without missing values, served as a reasonable benchmark for evaluating the performance of the Imp method. We set the missing proportion for CIs of 0%, 30%, or 60%. The missing mechanism was defined as missing completely at random (MCAR) or missing at random (MAR). The MAR mechanism was set so that the missing probability was dependent on the survival rate ( 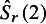 ): 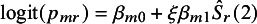 , where 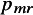 is missing probability, 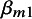 was set as the inverse of standard deviation of 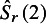 , 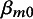 was numerically calculated so that the missing proportion would be at a set level, and 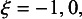 and 1. 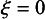 means the MCAR missing. In addition, 30% of the risk set size 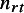 was missing from the MCAR missing mechanism. The Imp method is not used in settings where the missing proportion was 0%. This was set as the ideal situation for evaluating the performance of the Imp method.

In summary, we set 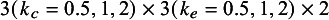 (dropout: 20%,40%) 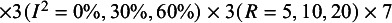 (missing proportion and mechanism: 0%, 30% ( 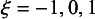 ), 60% ( 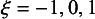 )) 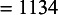 simulation conditions.

The number of simulations was 2,000, and a random-effects meta-analysis using the REML method for 1-, 2-, and 3-year survival rates was conducted with complete case analysis (CC) and the Imp method for each simulation. The Imp method was applied only when the missing proportion was not 0%. In each meta-analysis, point estimates and CIs for survival rates were estimated. No analysis was performed when the number of studies was less than 2 for the CC method. The performance evaluation indices were the simulation bias for the point estimate (on the original and log-log scales), the percentage bias for the SE (100({mean of SE} 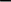 {SD of point estimates}) 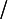 {SD of point estimates}) for the Imp method, the coverage proportion (CP) for the CIs, and the ratio of the mean SE for the Imp method to that for the CC method. We evaluated how the performance of the Imp method outperformed the CC method under missing conditions. Furthermore, we evaluated how the performance of the Imp method under missing conditions was close to the ideal results for conditions without missing. The latter was the more important evaluation. All random-effects meta-analyses, including case studies, were performed using the meta package^16^ in R ver. 4.4.3 (R Core Team, Vienna). The calculation program for 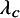 , 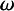 , 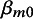 , 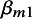 , the true values for 1-, 2-, and 3-year survival rates and the simulation program are given in the Supporting Information.

**Figure 1:**
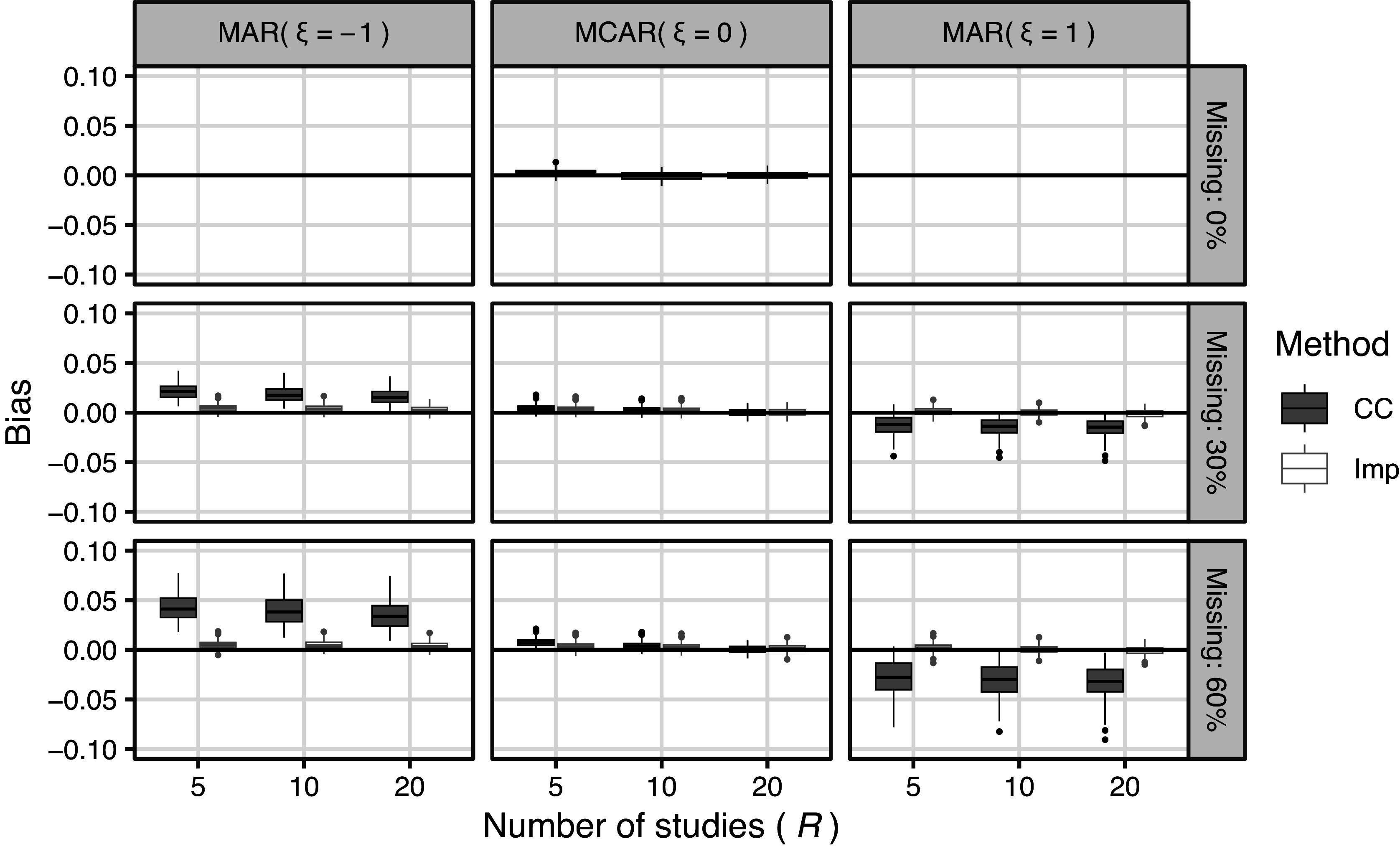
Results of simulation 4.1 (meta analysis): Simulation bias of survival rate for all settings.

**Figure 2:**
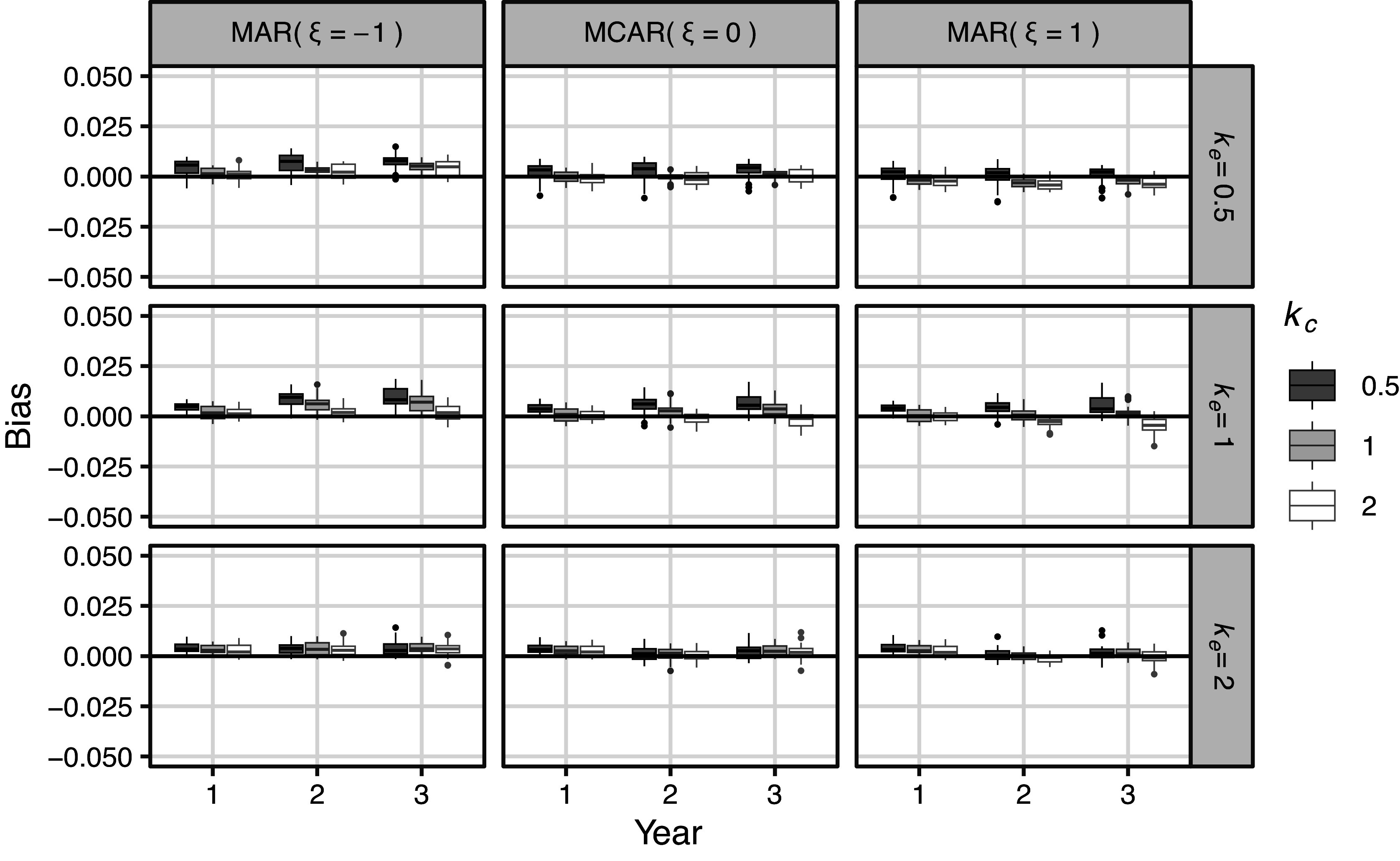
Results of simulation 4.1 (meta analysis): Simulation bias of survival rate for Imp method (missing proportion 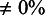 ).

#### 4.1.2 Simulation results

The simulation results are now described, with box-whisker plots showing the marginal distributions of the evaluation indices for each factor level that had a significant effect on the results of the prior evaluation. The full simulation results are available in the Supporting Information (Simulation_4.1_results.xlsx), which contains 1,134 (number of conditions) 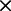 3 (number of years) 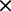 2 (Imp or CC) 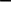 486 (Imp and missing proportion = 0%) = 6,318 lines. Figure 1 shows the simulation bias for all the settings. One box-whisker plot includes 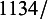 {7 (number of panels) 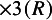 } 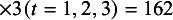 results. In the MCAR setting, the Imp method had a bias comparable to that of the CC method, which is a theoretically optimal analysis method in terms of bias. Under the MAR settings, the bias of the Imp method was substantially smaller than that of the CC method. Figure 2 illustrates the bias of the Imp method. The MAR had the greatest influence on bias in the Imp method. An estimator using the Imp method tended to have an overestimation bias when 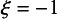 and underestimation bias when 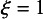 . Even when the shape of the distribution differed between events and censoring, when there was a deviation from the assumption, the bias did not increase significantly. The results for bias on the log-log transformed scale are provided in Figures S1 and S2 in the Supplementary Material. Apart from the reversal of sign due to the transformation, there were no major differences compared to the results on the original scale.

**Figure 3:**
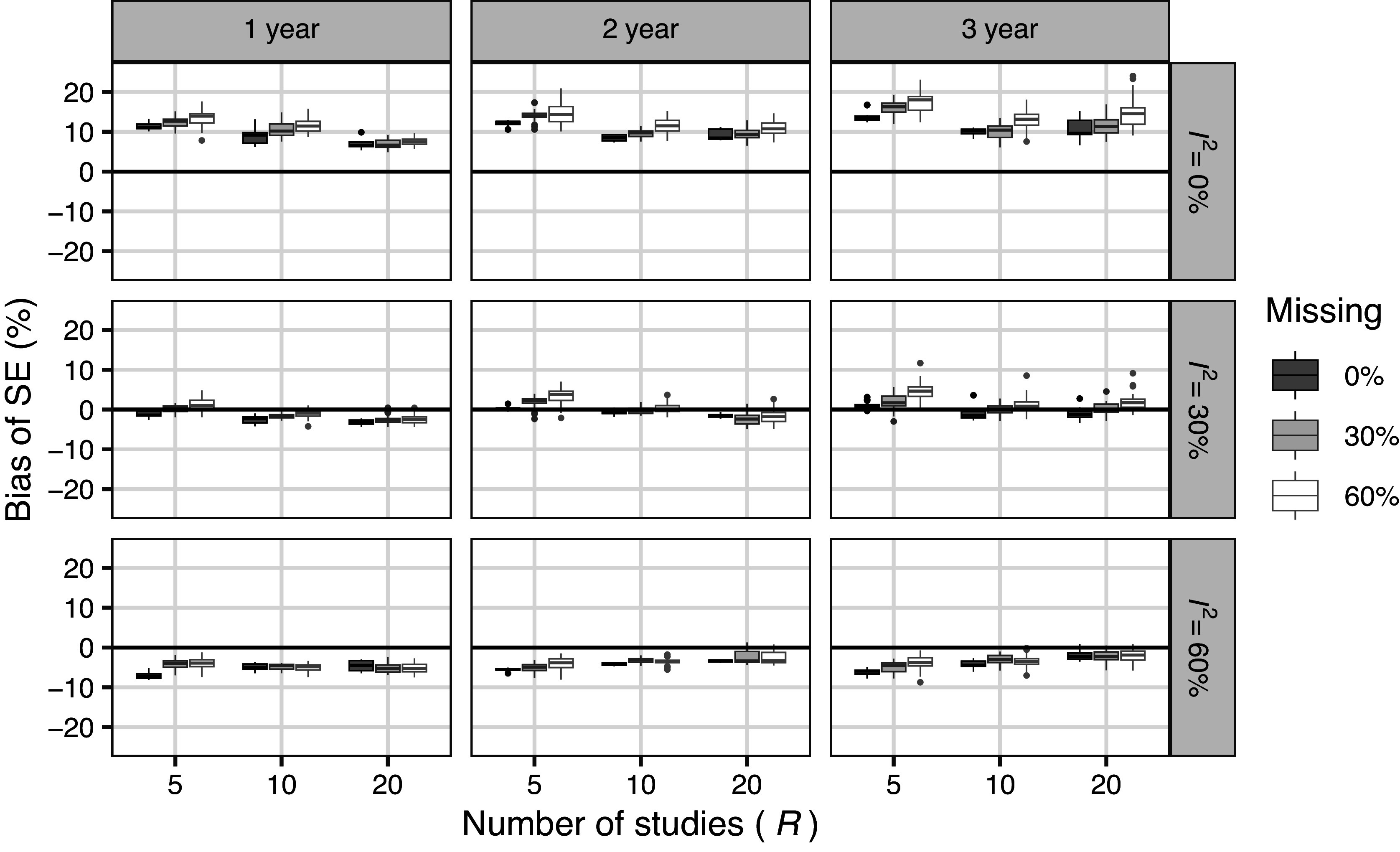
Results of simulation 4.1 (meta analysis): Simulation bias of SE for Imp method (including situation where missing proportion 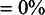 ).

Figure 3 presents the simulation bias for the SE of the Imp method. For reference, we have also included results with a 0% missing proportion. SE had an overestimation bias when 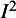 was small and an underestimation bias when 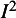 was large. This tendency was more apparent when the number of studies was small. Moreover, this tendency was independent of the proportion of missing SEs; in other words, it reflected the performance of the meta-analysis itself and not the proposed method. The underestimation bias did not worsen as the missing fractions increased. Compared with the results without missingness, the SE for the Imp method tended to be larger. This trend became stronger as the missing fraction increased.

**Figure 4:**
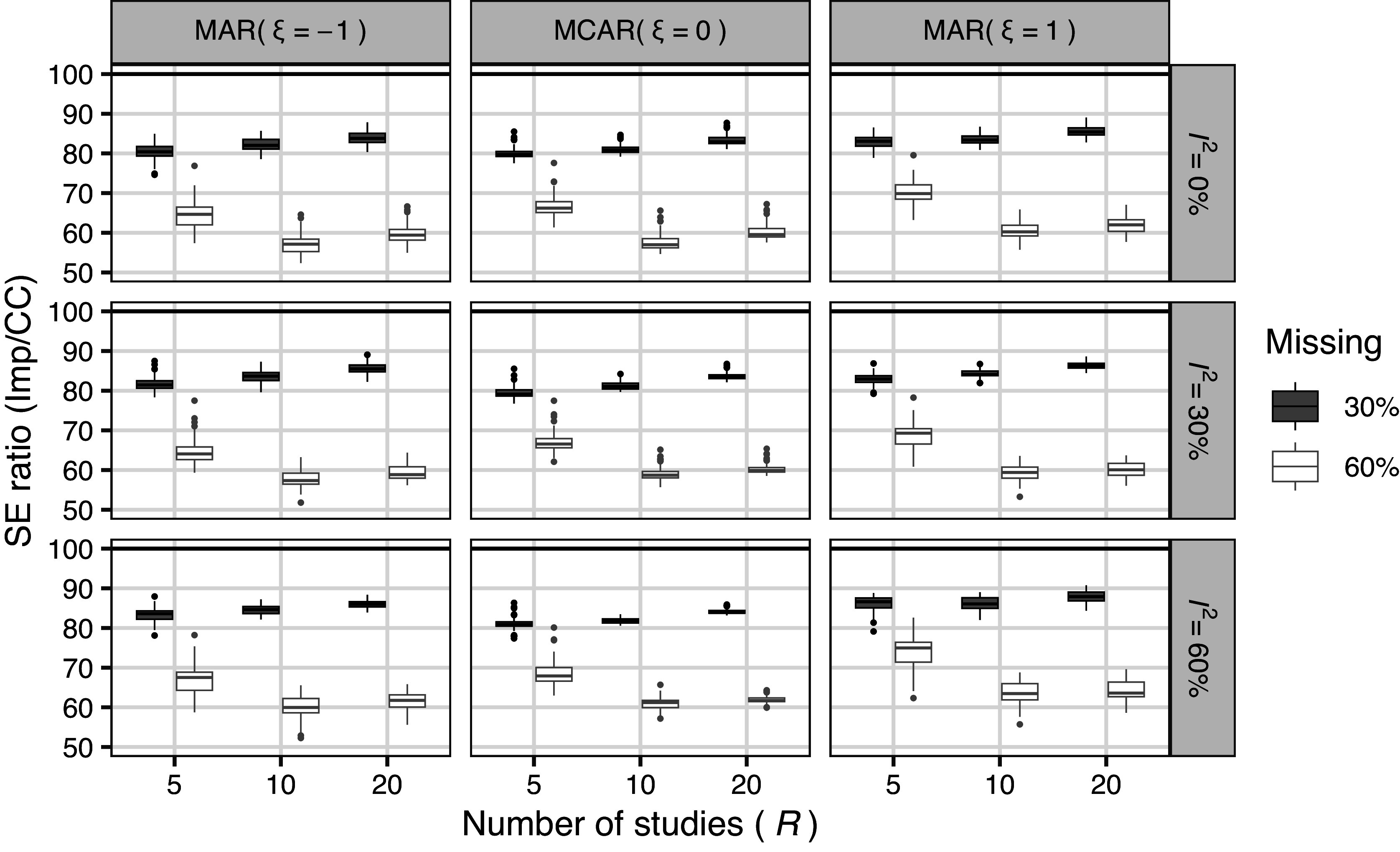
Results of simulation 4.1 (meta analysis): Ratio of simulated mean SE between methods (Imp/CC; missing proportion 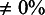 ).

Figure 4 shows the results for the ratio of the simulated mean SEs between the methods (Imp/CC). The SE of the CC method was significantly higher than that of the Imp method when the missing proportion increased. The ratio was close to 100 for 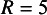 , which is due to the fact that no analysis was performed when the number of studies was less than 2 for the CC method.

**Figure 5:**
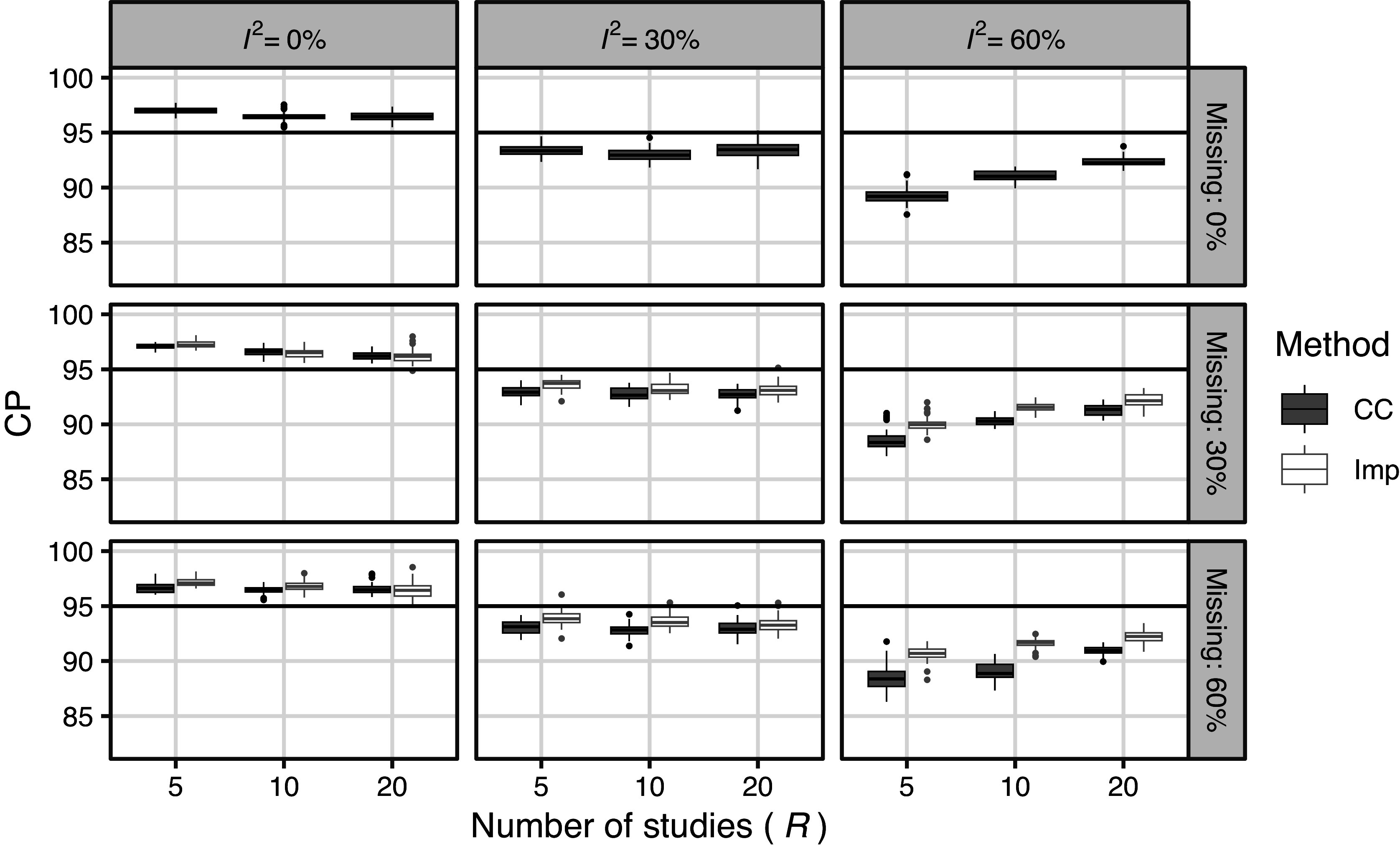
Results of simulation 4.1 (meta analysis): Simulated CP of CI for MCAR settings (including missing proportion 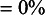 ).

Figure 5 shows the simulated CP of the CIs for the MCAR settings, including settings without missing data. First, CP was higher than the nominal level when 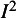 was zero, and lower when 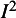 was large. This tendency was more pronounced when the number of included studies was small. These results were attributed to SE bias. In the case of MCAR, the CC method had a slightly lower CP than the Imp method, which was caused by the lower small-sample performance of the random effects meta-analysis. The results of the Imp method did not differ significantly from those obtained without missing measurements.

**Figure 6:**
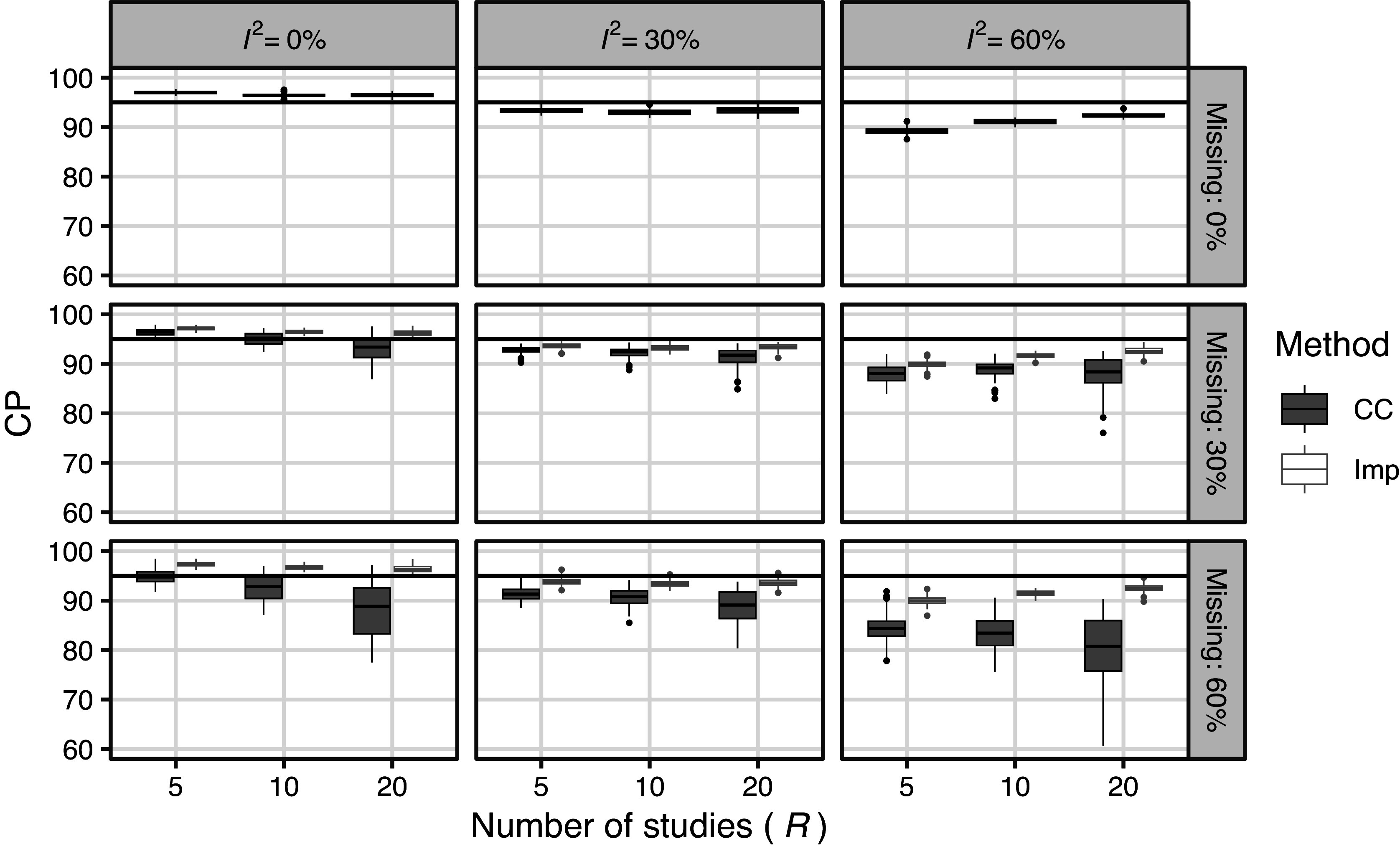
Results of simulation 4.1 (meta analysis): Simulated CP of CI for MAR settings (including missing proportion 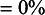 ). Some results of CC method exist below lower limit of graph.

Figure 6 shows the simulated CP of the CIs for the MAR settings; the results for the settings without missing data are included as a reference. For the CC method, CP became very low, mainly because of bias, particularly when *R* is large. For the Imp method, the CP could be slightly lower because of the bias in survival rates as *R* increased; however, the results were generally similar to those of cases without missing data.

### 4.2 Evaluation per study

#### 4.2.1 Simulation design

We evaluated the bias of the proposed SE for each study. The notation and settings are the same as those used for Simulation 4.1, but as it was a study-by-study evaluation, heterogeneity among the studies was not considered. The distributions for event and censoring were set as 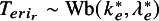 and 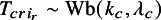 , respectively. We set 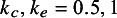 , or 2. We also set 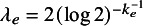 . 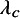 was numerically calculated such that the censoring proportion was 20% or 40%. We set 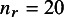 , 40, or 80.

Under each condition, 10,000 simulations were performed, and in each simulation the SEs on a log-log transformed scale of 1-, 2-, and 3-year survival rates were calculated. Furthermore, we computed the SEs based on the proposed method and calculated the mean difference from the actual SEs. We refer to this mean difference as “bias”. For the proposed method, SEs were calculated both when 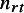 was available and missing.

Furthermore, the following additional indices were computed to assess what influences the bias of SE. For the proposed method with 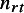 , we calculated the percentage bias of the life table estimator from the Kaplan–Meier estimator to check the influence of the assumption in Equation (4) and the percentage bias of 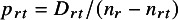 from true 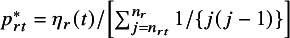 (from Equation (3)) to check the influence of the assumptions in equation (5), where 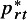 is a notation used as the true value. We also computed the percentage bias of the imputed (estimated) 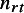 with the replacement of 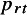 by 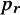 in equation (8) from the true 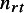 for the proposed method without 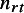 . The results of these additional indices are given in the Supplementary Material.

**Figure 7:**
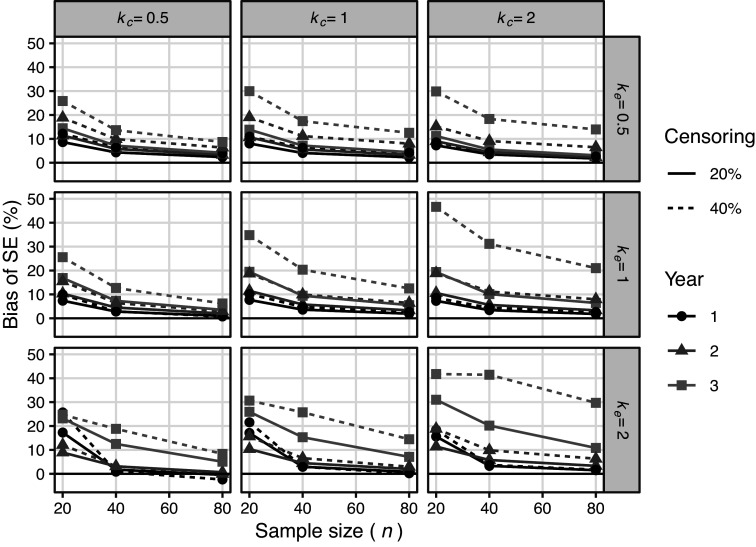
Results of simulation 4.2 (each study): Simulation bias of proposed SE with 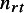 .

**Figure 8:**
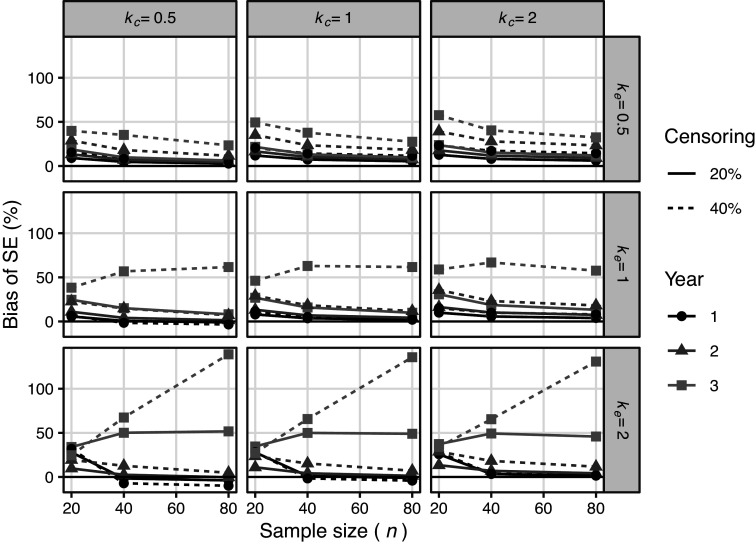
Results of simulation 4.2 (each study): Simulation bias of proposed SE without 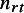 .

#### 4.2.2 Simulation results

Figure 7 shows the simulation results for the proposed SE with 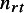 . The proposed method had over-estimation bias overall. The bias was large especially for small sample sizes, high censoring hazard, and post-median time points (3 years). The bias was approximately less than 20% in the other situations. These biases were caused by a combination of the bias in the life table estimator (Figure S3 in the Supplementary Material) and that in 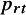 (Figure S4 in the Supplementary Material). The overestimation bias in the 3-year survival rate at high censoring rates was due to an overestimation bias of the life table estimator for 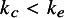 and that of 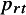 for 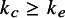 . The overestimation bias in the small sample was due to the bias of 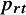 . Figure 8 shows the simulation results for the proposed SE without 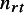 . The bias in the estimation of issues was large when 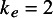 and the proportion of censoring was high, and the bias increased as the sample size increased. This was due to the under-estimation bias of 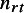 (Figure S5 in the Supplementary Material). In such cases, 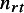 was frequently estimated to be less than 1 regardless of 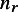 and replaced by 2. Therefore, the bias increased as the sample size increased.

**Figure 9:**
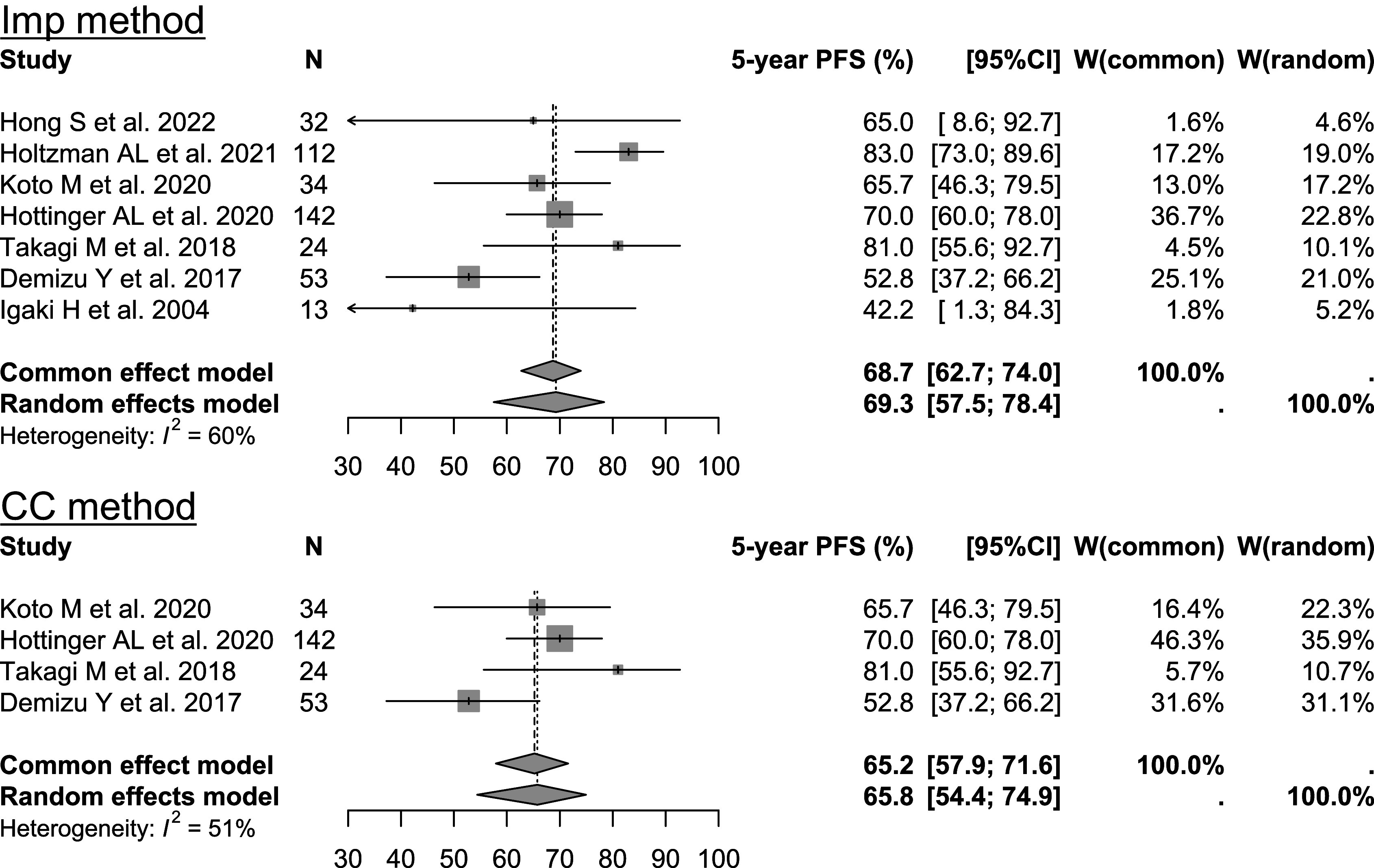
Results of meta analyses for skull base chordoma data. Imp: proposed imputation method, CC: complete case analysis method.

## 5 Case study

We applied our method to a systematic review of radiotherapy for skull base chordomas.^17^ Chordoma is a rare disease, with an incidence of 0.18 to 0.84 per million people.^18^ The common sites of occurrence include the sacrum and skull base, followed by the spine.^18^ As there are few randomized controlled studies in this research area, data from single-arm studies were collected and analyzed. We focused on particle beam therapy and performed a meta-analysis of the 5-year progression-free survival (PFS).^19^^–^ ^25^ Seven studies met the criteria in this systematic review, but only four reported CIs for 5-year PFS. For details on the systematic review, see Saito et al.^17^ The random-effects meta-analyses based on the REML method on the log-log transformed scale were conducted for these data using the Imp and CC methods. We also derived the results by applying the proposed method to four studies in which CIs were reported. Note that in the meta-analysis, the Imp method was used only in studies where SEs were missing. Detailed data and analyses are provided in the Supplementary Material (analysis_chordoma.R and analysis_chordoma.html).

**Table 1:** Results of SEs derived from reported CIs and SEs imputed with proposed method in chordoma data (log-log scale for SEs)

Study	*n*	5 year PFS	Derived SE	Imputed SE
Koto M et al. 2020	34	0.657	0.309	0.330
Hottinger AL et al. 2020	142	0.700	0.184	0.280
Takagi M et al. 2018	24	0.810	0.522	0.580
Demizu Y et al. 2017	53	0.528	0.222	0.193

Figure 9 presents the results of this analysis. Imputing the Imp method resulted in higher estimates based on the random-effects model compared with the CC method. The width of the CI for the Imp method was almost the same as that for the CC method, owing to the slightly larger 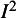 statistic of the Imp method. For reference, in the common-effects model, the widths of the CIs for the Imp method were much narrower than those for the CC method. Table 1 shows the results of SEs derived from the reported CIs and SEs imputed using the proposed method. While there was a slightly large overestimation of the SE in the study in the second row, the other studies did not deviate significantly from the values derived from the reported CIs.

## 6 Discussion

We developed a new method to impute missing precision information (CIs) in meta-analyses of survival rates. The simulation results showed that excluding studies with missing precision information could cause serious bias and precision loss in random-effects meta-analyses for survival rates. We also found that the Imp method significantly reduced bias and improved precision compared to cases that excluded studies with missing precision information. Note that in MAR situations, where the probability of missing precision information depends on high survival rates, there is a risk that estimators based on the proposed method might have a small bias. In addition, our proposed method relies on several assumptions; however, based on simulations, it was shown to be less sensitive to deviations from these assumptions. Our simulations also showed that there was no underestimation bias in the SE owing to the single-imputation approach of our proposed method. In the simulations, we used a random-effects model. However, the proposed method can be used in a manner similar to a fixed-effects model.

In cases where only a small number of studies remain after excluding those with missing precision information, the CC method suffers from low-precision and generalizability problems. Even when the number of studies was small, the CC method may have caused a significant decrease in CP owing to bias. Therefore, it is recommended that the proposed method be applied in both cases.

The proposed method performed well in the meta-analysis results (Simulation 4.1). In contrast, an overestimation bias was observed in each study (Simulation 4.2), especially for small sample sizes, high censoring rates, and 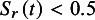 (3-year survival). For high censoring rates, 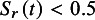 (3-year survival), and a wear-out failure type event hazard 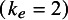 , the SE imputation method without 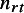 had a large overestimation bias. Thus, caution should be exercised when 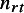 is not obtained. However, this does not imply that such studies should be excluded without using the proposed method. Excluding studies with missing precision information (i.e., the CC method) is equivalent to estimating the SE of such studies to be infinite. It should be noted, however, that in almost all cases, the SE of the proposed method did not have an under-estimation bias. Therefore, the proposed method will, on average, handle missing information more appropriately than the CC method for each study.

The limitations of this study were as follows: This study did not cover missing estimates of the survival rate. In such cases, it may be possible to extend the proposed method to multivariate meta-analysis. If there is variation between studies in whether 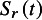 is reported, it can be inferred that 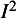 is probably very large. For example, there may be a certain percentage of studies that are not followed until survival at a particular time point can be estimated, or all cases may have had an event by a particular time point. In such cases, one should be careful in interpreting the results of the meta-analysis. The case of 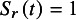 was also addressed empirically. If the true value of 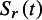 is close to 1, the proposed method may have a bias. Therefore, it should be noted that the performance of the proposed method for 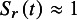 has not been sufficiently verified. Another approach is to identify a Weibull distribution that fits the Kaplan–Meier plot and estimate 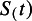 parametrically. Furthermore, although several other methods exist for the estimation of confidence intervals for the Kaplan–Meier estimator, we did not deal with any method other than those used as default settings in the major software packages. The performance of the random-effects meta-analysis of survival rates was poor, especially in terms of CP, when the number of studies was small and the heterogeneity was high. This seems to be due to the fact that the ordinary random-effects model is misspecified because the data generation structure is not based on a normal distribution. Although the random-effects model is asymptotically valid because of the asymptotic normality of the survival rate estimator, its performance is not guaranteed for small samples, which may explain the somewhat lower performance when the number of studies is small. Improvements in the inference method and the model itself may be necessary. When information on 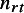 was missing, SE based on the proposed method was at risk of having a large overestimation bias when deviating from the assumption that 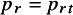 . Improving the performance of the proposed method when 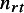 is missing is an issue for future study. In the case example, the proposed method increased heterogeneity and leads to wider CIs compared with those of the CC method. This may seem to contradict the claim that the method improves precision; however, the missing CIs may have excluded studies that should have induced high heterogeneity. In such case, the CC method would have resulted in inappropriately low heterogeneity. Although the true structure of the case study is unknown, the usefulness of the proposed method should be further examined by applying the proposed method to more real data in the future. In this study, we did not deal with methods related to the restoration of pseudo-IPD. Comparison of the proposed method with these methods and development of a hybrid method between the pseudo-IPD method and proposed method will be the subject of future research. In the meta-analysis, we focused only on the log-log transformation method as a unified scale. However, Hosmer and Lemeshow^12^ claim that if a software package’s default is the log, log-log, or logit transformation method, there is little practical reason for the switch to a different transformation. Therefore, it would not be problematic to use the log transformation method among the three methods considered in this study, which is the default setting in the survfit function in R. Other methods such as the logit transformation method are not discussed in this study.

Addressing these issues is a topic for future research.

## Supporting information

Maruo et al. supplementary material Maruo et al. supplementary material

## Data Availability

The chordoma data set is openly available from metaSurvMissCI package (see Appendix).

## Supplementary Material

The supplementary material for this article can be found at https://doi.org/10.1017/rsm.2025.10024.

## Appendix: Software

The R package, metaSurvMissCI, was developed to implement the Imp method (https://github.com/kzkzmr/metaSurvMissCI). The developed version of metaSurvMissCI from GitHub with: devtools::install_github("kzkzmr/metaSurvMissCI"). If the devtools package has not been installed, please install it.

The metaSurvMissCI package includes two functions, impute_se_surv and forest_surv. The impute_se_surv function imputes missing precision information for the meta-analysis of survival rates and calculates survival rates and their SEs on the transformed scale. This function also implements the procedure described in Section 2.2 for transforming the results of studies with reported precision information onto a unified scale. The forest_surv function provides a forest plot of the meta-analysis of survival rates using the data frame returned by the impute_se_surv function. To obtain more information on each function, type ?impute_se_surv, and ?forest_surv. The sample program for the case study is a good illustration of this package.
