# Supplementary material for: Simple imputation method for meta-analysis of survival rates when precision information is missing
Source: Res Synth Methods. 2025 Sep 11;16(6):937–52. doi: 10.1017/rsm.2025.10024 (PMC12657670; doi:10.1017/rsm.2025.10024)
Supplement: Maruo et al. supplementary material [file S1759287925100240sup001.zip › Supporting_information_Rcode_Simulation_results/analysis_chordoma.html]

Meta analysis for chordoma data


# Meta analysis for chordoma data

#### Kazushi Maruo

```
#devtools::install_github("kzkzmr/metaSurvMissCI") # install for the first time
library(metaSurvMissCI)
library(meta)
impdata <- impute_se_surv(data = metadata_chordoma, St = "PFS5y",
                          LCL = "PFSL5y", UCL = "PFSU5y", n = "n",
                          nt = "n_5yPFS", ne = "ne_PFS", p = "pr_PFS")
meta_imp <- metagen(TE = tr_St, seTE = tr_SE, studlab = Study,
                    data = impdata, method.tau = "REML")

meta_cc <- metagen(TE = tr_St, seTE = tr_SE, studlab = Study,
                   data = subset(impdata, imputed == 0), method.tau = "REML")
```

# Meta analysis for 5 year PFS

## Data imputed with the proposed method

```
#svg("Imp_case.svg", width = 9, height = 3.1)
forest_surv(meta_imp, xlim = c(30, 100), estlab = "5-year PFS (%)",
            print.tau2 = FALSE, print.pval.Q = FALSE)
```

```
#dev.off()
```

## Data without missing confidence intervals

```
#svg("CC_case.svg", width = 9, height = 2.5)
```

```
forest_surv(meta_cc, xlim = c(30, 100), estlab = "5-year PFS (%)",
            print.tau2 = FALSE, print.pval.Q = FALSE)
```

```
#dev.off()
```
