# Supplementary material for: Simple imputation method for meta-analysis of survival rates when precision information is missing
Source: Res Synth Methods. 2025 Sep 11;16(6):937–52. doi: 10.1017/rsm.2025.10024 (PMC12657670; doi:10.1017/rsm.2025.10024)
Supplement: Maruo et al. supplementary material [file S1759287925100240sup001.zip › Supporting_information_figure.pdf]

# Supporting information for ”Simple imputation method for meta-analysis of survival rates when precision information is missing”

Kazushi Maruo  Yusuke Yamaguchi  Ryota Ishii  Hisashi Noma
Masahiko Gosho

## Additional figures for simulation

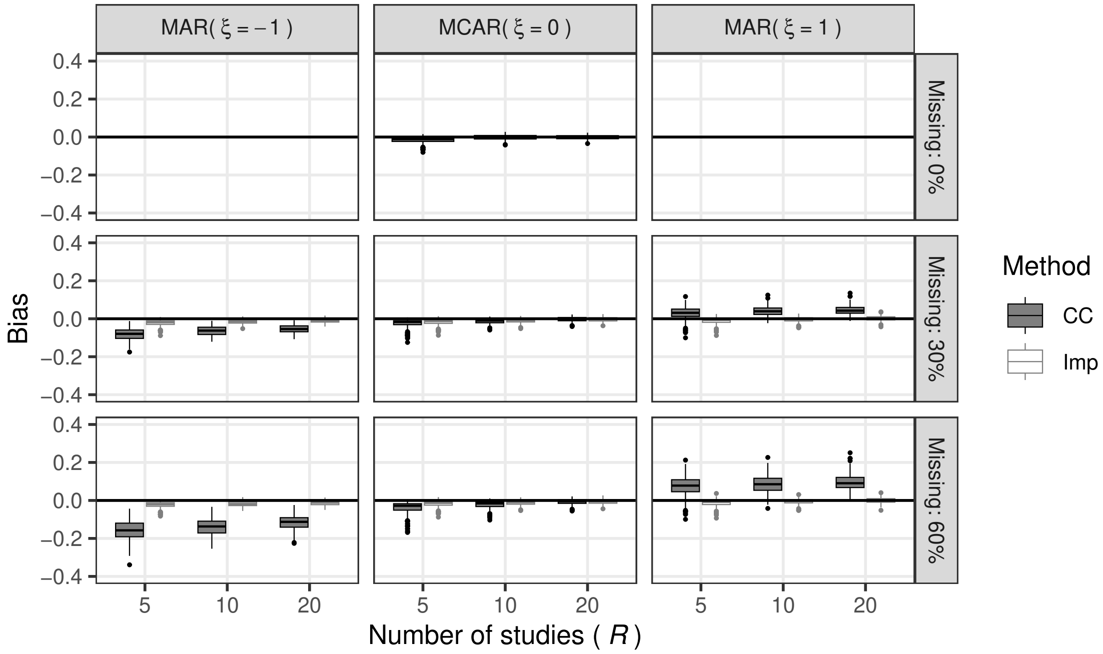


Figure S1: Results of Simulation 4.1 (meta analysis): Simulation bias of survival rate on log-log scale for all settings.

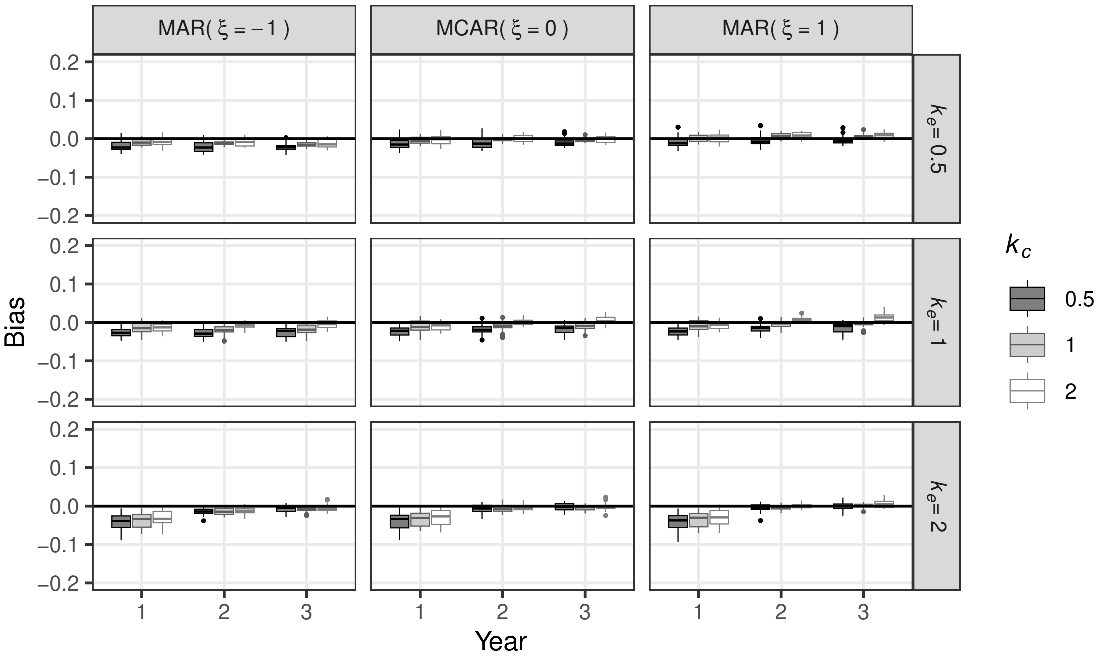


Figure S2: Results of Simulation 4.1 (meta analysis): Simulation bias of survival rate on log-log scale for proposed (Imp) method (missing proportion $\neq 0\%$).

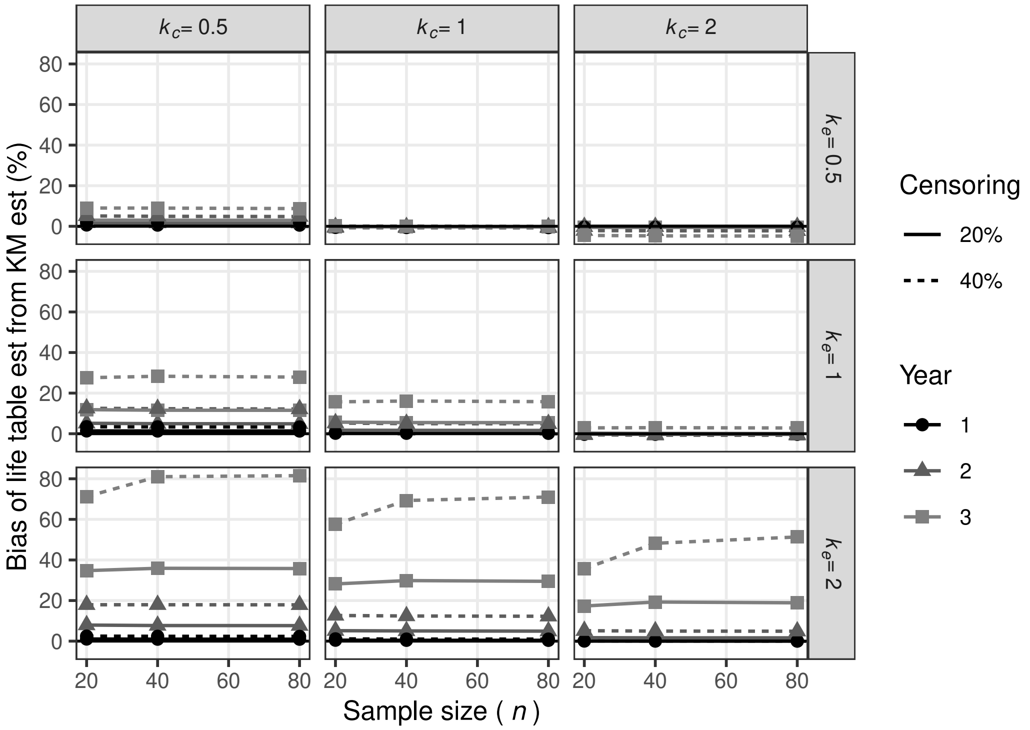


Figure S3: Results of Simulation 4.2 (each study): Bias of lifetable estimator from Kaplan-Meier estimator.

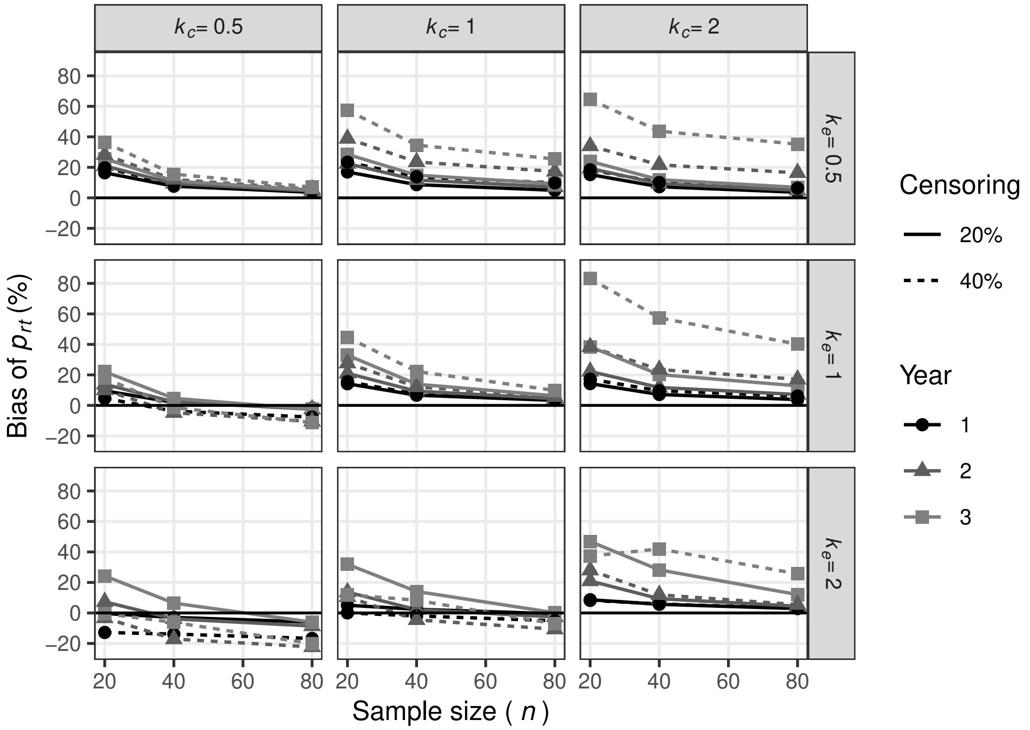


Figure S4: Results of Simulation 4.2 (each study): Bias of $p_{rt}$.

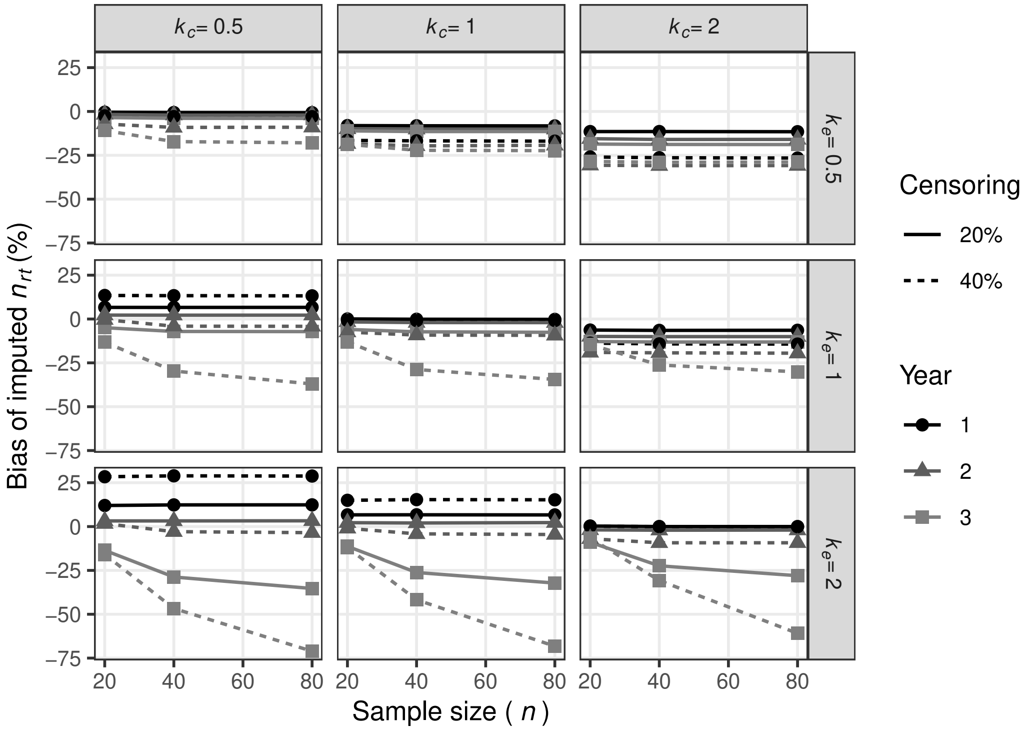


Figure S5: Results of Simulation 4.2 (each study): Bias of imputed $n_{rt}$.
